# Predictive analysis of osteoarthritis and chronic pancreatitis comorbidity: complications and risk factors

**DOI:** 10.3389/fendo.2024.1492741

**Published:** 2024-11-06

**Authors:** Iryna Halabitska, Pavlo Petakh, Valentyn Oksenych, Oleksandr Kamyshnyi

**Affiliations:** ^1^ Department of Therapy and Family Medicine, I. Horbachevsky Ternopil National Medical University, Ternopil, Ukraine; ^2^ Department of Biochemistry and Pharmacology, Uzhhorod National University, Uzhhorod, Ukraine; ^3^ Broegelmann Research Laboratory, Department of Clinical Science, University of Bergen, Bergen, Norway; ^4^ Department of Microbiology, Virology, and Immunology, I. Horbachevsky Ternopil National Medical University, Ternopil, Ukraine

**Keywords:** osteoarthritis, chronic pancreatitis, adults, elderly, cohort study

## Abstract

**Background:**

The comorbidity of chronic pancreatitis (CP) in patients with osteoarthritis (OA) is insufficiently studied, and the reciprocal impact of these conditions remains poorly understood. This study aimed to investigate potential predictors for the development of CP in OA patients, as well as associated complications.

**Methods:**

A cohort of 181 patients was categorized into four groups: a control group (n=30), patients with OA (n=68), patients with CP (n=31), and patients with OA and comorbid CP (n=52). All four groups had no statistical differences in age and gender. The study utilized the WOMAC index, Visual Analog Scale (VAS), Lequesne index, biochemical assays, and advanced statistical methods to assess joint status in OA patients with comorbid CP. It explored potential predictors of comorbidity development and associated complications.

**Results:**

The study revealed that concurrent CP in OA exacerbates progression and contributes to malnutrition. Body Mass Index (BMI) emerged as a potential predictor for CP comorbidity development in OA patients. Factors such as the WOMAC total score, fecal elastase-1, C-reactive protein (CRP), ferritin, retinol, tocopherol, 25-hydroxyvitamin D3, and BMI were found to influence the development of comorbidity of CP in OA. Additionally, Gastrointestinal Symptom Rating Scale-Diarrhea Syndrome (GSRS-DS), Gastrointestinal Symptom Rating Scale-Constipation Syndrome (GSRS-CS), Qualitative Assessment of the Symptoms and Impact of Pancreatic Exocrine Insufficiency Domain A (PEI-Q-A), retinol, tocopherol, and iron were identified as potential predictors comorbidity CP with exocrine pancreatic insufficiency in OA patients.

**Conclusion:**

The presence of CP in OA patients exacerbates disease progression and complications, necessitating further investigation.

## 1 Introduction

Osteoarthritis (OA) and chronic pancreatitis (CP) are both prevalent chronic conditions that pose significant health challenges and often interact in complex ways (1). OA, a leading cause of disability in the elderly, predominantly affects the hips, knees, and hands, and early diagnosis of cartilage lesions through methods like X-ray, ultrasound, and MRI is crucial for effective treatment and preservation of joint function (2). OA affects around 27% of individuals over 45 years old, primarily impacting peripheral synovial joints, and is the most common form of arthritis (3). Osteoarthritis is primarily regarded as a condition affecting older individuals, with over one-third of those aged 65 and older exhibiting OA in at least one joint (4). The link between obesity and knee osteoarthritis, with obesity as a key risk factor, is well-established (5). It imposes a considerable economic and social burden, particularly on women, and is expected to increase due to aging populations and rising obesity rates (6). Knee OA causes joint pain worsened by use and relieved by rest, with nonpharmacologic and pharmacologic treatments managing symptoms but not reversing the disease, and surgery considered only when these measures fail to control pain (7, 8). Obesity, sarcopenia, and sarcopenic obesity are prevalent among patients with end-stage knee osteoarthritis (9). Frailty, characterized by physical decline in later life and linked to negative health outcomes, may be influenced by the presence of knee OA (10). A clinical trial found that a 48-month weight loss and exercise program may reduce the risk of knee OA in at-risk adults compared to controls (11). Despite the high prevalence, OA lacks disease-modifying treatments, with management focusing on analgesics, physical therapy, and surgical options (12). Chronic pain and disability associated with OA are exacerbated by persistent inflammation, which further damages joints (13). If topical treatment is ineffective or impractical, therapy generally consists of an oral NSAID with a proton-pump inhibitor or a COX-2 inhibitor, tailored to the patient’s gastrointestinal, cardiovascular conditions, and pain extent (14). Recent research suggests that neuroinflammatory processes may contribute to chronic pain and mood disorders in OA, highlighting potential new therapeutic targets (6).

Chronic pancreatitis (CP), marked by recurrent pancreatic inflammation and fibrosis, leads to progressive dysfunction of pancreatic functions and severe complications like pancreatic insufficiency, diabetes, and an increased risk of cancer. It is often associated with alcoholism but is also influenced by genetic and environmental factors (15). CP results in chronic abdominal pain, impaired digestion, malnutrition, and heightened risks of infections and psychiatric disorders (16, 17).

The rising incidence of CP and the absence of a definitive cure necessitate advanced imaging and a multidisciplinary approach for effective management (18, 19). OA and CP present with comorbid conditions, including diabetes, osteoporosis, and psychiatric issues, requiring integrated and coordinated care (3, 20). The intersection of OA and CP underlines the need for comprehensive management strategies to address their combined effects on patient health, emphasizing the importance of addressing overlapping conditions and complex care needs (12, 18).

The OA and CP comorbidity has not been adequately studied, and the mutual influence of these conditions is poorly understood. Existing research does not provide sufficient data on the interactions and shared pathophysiological mechanisms of OA and CP. Therefore, further investigation is essential to clarify the reciprocal effects of these diseases and to develop more effective treatment approaches.

The hypothesis of our study aimed to investigate the characteristics of comorbid osteoarthritis and chronic pancreatitis, focusing on the effects of osteoarthritis on the indicators of exocrine pancreatic insufficiency and the resultant disturbances in nutritional status, as well as the influence of chronic pancreatitis on the progression and course of osteoarthritis. Furthermore, we seek to identify predictors that facilitate the development of comorbidity between osteoarthritis and chronic pancreatitis, thereby increasing the risk of complications.

## 2 Materials and methods

### 2.1 Subjects

The study included 181 patients, who were divided based on the type of pathology being studied and the presence of comorbidities. The control group consisted of 30 practically healthy patients who did not have chronic musculoskeletal and digestive diseases. The second group included 68 patients with osteoarthritis. The third group comprised 31 patients with chronic pancreatitis. The fourth group included 52 patients with comorbid OA and CP. All four groups had no statistical differences in age (p = 0.7145) and gender (p = 0.8716). There was also no statistical difference found between the duration of OA in the second and fourth groups (p = 0.069). No statistical difference was found between the duration of chronic pancreatitis in the third and fourth groups (p = 0.281) (**Table 1**). The initial demographic characteristics showed no statistically significant differences between the four groups. The patients included in the study did not abuse alcohol. According to the CAGE questionnaire, all patients scored less than 2 points. None of them were diagnosed with gallstone disease.

**Table 1:** Age, gender characteristics, and duration of OA in patients included in the study.

	Control	OA	CP	OA+CP	p-value ^a^
	(n=30)	(n=68)	(n=31)	(n=52)	
Male	53.3%	55.9%	54.8%	53.8%	p = 0.8716
Female	43.7%	44.1%	45.2%	46.2%	
Age	43 (40–50)	45 (38.75–51.25)	44 (37-53)	44 (38–51)	p = 0.7145
Duration of OA	0 (0-0)	7 (5–9)	0 (0-0)	8 (6–9)	p = 0.069
Duration of CP	0 (0-0)	0 (0-0)	7 (4.5-9.5)	7.5 (5–9)	p = 0.281

Median and interquartile range (IQR) were used to summarize the data. ^a^Kruskal–Wallis test.

Criteria for inclusion of patients in the study comprised individuals of both genders; confirmed diagnosis of hip and knee osteoarthritis (based on the International Classification of Diseases, 10th Revision codes M16, M17); and confirmed diagnosis of CP (based on the International Classification of Diseases, 10th Revision code K86). Exclusion criteria for the study included: Zollinger-Ellison syndrome, Shwachman syndrome, Johanson-Blizzard syndrome, Clark-Hedvild syndrome, history of pancreatic resection, pancreatic tumors, large pancreatic cysts, subcompensated and decompensated type II diabetes mellitus, type I diabetes mellitus, stomach malignant tumors, post-gastrectomy status, stomach and duodenum peptic ulcer, dumping syndrome, post-cholecystectomy status, gallstone disease, malignant liver tumors, viral hepatitis, liver cirrhosis, cystic fibrosis, non-specific ulcerative colitis, Crohn’s disease, celiac disease, decompensated heart-lung diseases, thyroid gland pathology, arrhythmias, stage II-III hypertension, unstable ischemic heart disease, acute myocardial infarction, recent major surgery within the last month, use of systemic glucocorticosteroids, stage III-V chronic kidney disease, pregnancy, severe exhaustion, tendency to bleed, suspicion of malignant tumors, psychiatric and behavioral disorders, infectious and parasitic diseases, congenital anomalies and chromosomal disorders, and refusal to participate in the study.

Participants for this study were enrolled from the Ternopil City Communal Institution “Center for Primary Medical and Sanitary Care” between 2019 and 2023. The research adhered to the fundamental principles outlined in the Council of Europe’s Convention on Human Rights and Biomedicine, and was conducted in accordance with the ethical guidelines specified in the World Medical Association’s Declaration of Helsinki regarding medical research involving human subjects, including subsequent revisions. Furthermore, it complied with Ministry of Health of Ukraine Order No. 690 dated September 23, 2009. All participants provided informed consent prior to their involvement in the study. Approval for the study was obtained from the Bioethics Committee of I. Horbachevsky Ternopil National Medical University, Ministry of Health of Ukraine (Protocol No. 75, November 1, 2023). The study cohort consisted of individuals of Ukrainian ethnicity with European ancestry, including adults aged ≥18 years and the elderly aged ≥60 years (**Table 1**).

The diagnosis of OA was established according to international recommendations (21). Joint examination included inspection, palpation, objective assessment of pain at rest and movement. Radiological stages of OA were assessed according to the classification by J.H. Kellgren and J.S. Lawrence. MRI findings were also considered in the analysis.

The diagnosis of CP was verified according to international recommendations (22).

The CAGE questionnaire was used to assess the patients’ predisposition to alcohol abuse.

### 2.2 Laboratory and clinical data

In evaluating the joint status among individuals afflicted with OA, the WOMAC index (Western Ontario and McMaster Universities Osteoarthritis Index) was a pivotal assessment tool (23, 24). Meanwhile, to appraise the severity of OA in the study cohort, researchers employed the Lequesne algofunctional index (24). To evaluate pain, range of motion, and functional impairment in affected joints, it was employed the Visual Analog Scale (VAS) (25). For the assessment of fecal α-elastase levels, enzyme-linked immunosorbent assay (ELISA) employing standard proprietary kits was employed (BIOSERV Diagnostics Gmbh (Germany)).

The colorimetric method was used to determine the level of bilirubin. Alanine aminotransferase (ALT) and aspartate aminotransferase (AST) levels were assessed using the Reitman-Frankel method. Gamma-glutamyl transferase (GGT) and alkaline phosphatase levels were measured using the kinetic colorimetric detection method.

The PEI-Q (Qualitative Assessment of the Symptoms and Impact of Pancreatic Exocrine Insufficiency) questionnaire was employed to evaluate Exocrine Pancreatic Insufficiency (EPI) (26). The Gastrointestinal Symptom Rating Scale (GSRS) questionnaire was employed to assess gastrointestinal symptoms (27).

Retinol, tocopherol, thiamine, and pyridoxin levels were quantified using spectrophotometric methods and respective assay systems. 25-hydroxyvitamin D (25-OHD) levels were determined using enzyme-linked immunosorbent assay (ELISA) kits specific for 25-OHD (BIOSERV Diagnostics Gmbh (Germany)).

The Body Mass Index (BMI) was calculated by taking an individual’s weight in kilograms and dividing it by the square of their height in meters. Heights of participants were measured in meters, while their weights were recorded in kilograms. Based on the resulting BMI values, individuals were classified into the following categories: underweight for a BMI less than 18.5, normal weight for a BMI ranging from 18.5 to 24.9, overweight for a BMI between 25.0 and 29.9, and obesity for a BMI of 30.0 or higher (28).

The concentrations of red blood cells (RBC), white blood cells (WBC), and hemoglobin were assessed using an automated hematology analyzer. Serum ferritin levels were quantitatively assessed using the Ferritin Audit Diagnostics reagent kit and an enzyme-linked immunosorbent assay (ELISA) method.

Transferrin levels in serum were quantified using an automated assay method employing Transferin Audit Diagnostics reagents and an enzyme-linked immunosorbent assay (ELISA) method [BIOSERV Diagnostics Gmbh (Germany)].

The concentration of C-reactive protein (CRP) was assessed using the latex turbidimetric method.

### 2.3 Statistical analysis

Patient demographics and clinical data underwent thorough evaluation and were reported using descriptive statistics. The Shapiro-Wilk test was used to assess the normality of the data distribution. Because the data were not normally distributed, medians and interquartile ranges were computed for all variables. A statistical significance level (p) of less than 0.05 was employed for hypothesis testing.

The Mann-Whitney U test was applied to assess differences between two independent groups. For comparisons involving three or more groups, the Kruskal-Wallis test was employed. Subsequently, Dunn’s multiple comparison test was performed to evaluate pairwise differences between groups in *post hoc* analysis.

Spearman’s rank correlation coefficient was computed to examine the associations among continuous variables as part of a correlation matrix analysis.

The diagnostic significance of predictors for the presence of comorbidity between osteoarthritis and CP, as well as the development of exocrine pancreatic insufficiency in osteoarthritis, was evaluated using ROC curves (Receiver Operating Characteristic). This analysis included the calculation of ROC metrics such as the Area Under the ROC Curve (AUC) with a 95% confidence interval (CI), the Youden index (J), associated cutoff point, sensitivity (Se), and specificity (Sp). Sensitivity demonstrated the test’s capacity to accurately detect individuals with the condition, correctly identifying true positives while minimizing the number of missed diagnoses. Specificity, on the other hand, indicated the test’s effectiveness in identifying those without the condition, reducing false positives and ensuring precise exclusion of healthy individuals.

Binary logistic regression was employed to identify potential predictors associated with the development of exocrine pancreatic insufficiency in osteoarthritis patients.

Principal component analysis (PCA) was conducted to identify factors related to the comorbidity of osteoarthritis and CP.

Statistical analyses were conducted using commercially available software packages, including IBM SPSS Statistics (version 25).

## 3 Results

### 3.1 Comparing group expression

The indicators of OA course were investigated in a group of patients with OA, CP, and in the context of comorbidity between OA and CP. No significant difference in the Kellgren-Lawrence grade of OA was observed between the group of patients with OA and the group of patients with the comorbidity of OA and CP (p = 0.1437). It was established that the WOMAC indices for pain (p < 0.001), stiffness (p = 0.02), function (p < 0.001), and total (p < 0.001) were higher in the group of patients with comorbidity of OA and CP compared to the group of patients with OA. In the examination of the Lequesne Algofunctional Index, it was noted that this measure exhibited greater values in the cohort of patients experiencing comorbidity compared to those with OA alone (p < 0.001). Furthermore, it was observed that the presence of both OA and CP led to elevated scores in various indices, including VAS assessments for joint pain, movement, inflammation, and joint dysfunction, in comparison to the group solely diagnosed with OA (p < 0.001) (**Table 2**).

**Table 2:** Indicators of OA course.

	OA (n=68)	OA+CP (n=52)	p-value ^a^
Kellgren-Lawrence grade	1.5 (1-2)	2 (1-2)	p = 0.1437
WOMAC pain	10 (10-11)	16 (16-16)	p < 0.001
WOMAC stiffness,	3 (3-3)	4(4-5)	p = 0.02
WOMAC function	28 (28-29)	38 (37-39)	p < 0.001
WOMAC total	42 (41-43)	58 (57.75-60)	p < 0.001
Lequesne Algofunctional Index	5 (5-6)	8 (8-8)	p < 0.001
VAS joint pain	31 (30-32.25)	44 (43-45)	p < 0.001
VAS movement	37 (36-38.25)	52 (51-52)	p < 0.001
VAS inflammation	16 (15-17)	28 (27-29)	p < 0.001
VAS joint dysfunction	15 (14-16)	30 (29-31)	p < 0.001

Median and interquartile range (IQR) were used to summarize the data. ^a^Mann–Whitney test.

Examination of fecal elastase-1 levels revealed significant differences among the patient groups studied (p < 0.001). Levels of fecal elastase-1 were found to be lower compared to the control group (p < 0.001). Additionally, patients with comorbid OA and CP showed a decrease in fecal elastase-1 levels compared to those with OA (p < 0.001) and CP (p = 0.0465) (**Supplementary Table S1**).

No significant differences were observed in the levels of total bilirubin (p = 0.1767), direct bilirubin (p = 0.1767), indirect bilirubin (p = 0.6505), ALT (p = 0.0787), AST (p = 0.1886), GGT (p = 0.7773), and alkaline phosphatase (p = 0.0633) among the groups under study. No statistically significant differences were found between the groups in the *post hoc* analysis.

The PEI-Q scale indicators analysis detected abdominal and bowel movement symptoms among patients with OA as per the questionnaire; nevertheless, the overall symptom score did not surpass 0.6 points. Furthermore, PEI-Q scores were higher in patients with CP and comorbid OA and CP compared to those with OA alone (p < 0.001). A statistically significant difference was also found between all groups based on the PEI-Q questionnaire scores (**Supplementary Table S1**).

The presence of gastrointestinal symptoms was established in the group of patients with OA according to the GSRS questionnaire scales. However, the levels of indicators on all scales of the GSRS questionnaire were higher in patients with comorbidity of OA and CP compared to the group of patients with OA (p < 0.001). No statistically significant difference was found between the groups of patients with CP and with comorbid OA and CP with respect to the DS and CS scales of the GSRS, for other indicators, a statistically significant difference was observed. The highest level of scores in both groups of patients was on the abdominal pain scale of the GSRS questionnaire (**Supplementary Table S1**).

Examination of fat-soluble vitamins retinol, tocopherol, and 25(OH) vitamin D3 levels revealed significant differences among all studied groups (p < 0.001). Patients of groups with OA and CP without comorbidity exhibited lower levels of these fat-soluble vitamins compared to the control group (p < 0.001). Furthermore, a reduction in the levels of all examined vitamins was noted in patients with comorbid OA and CP compared to those with OA alone (p < 0.001). No statistically significant difference was found in the levels of retinol and tocopherol between the groups with CP and with comorbid OA and CP (**Supplementary Table S2**).The study found no significant differences in the levels of thiamine (p = 0.6889) and pyridoxin (p = 0.1408) among the groups.

Upon analysis of BMI, varying levels of this parameter were identified across all investigated groups (p < 0.001). The OA group had the highest BMI (p < 0.001). In the cohort with comorbid OA and CP, BMI was lower than in the OA group (p < 0.001) but higher compared to the control group (p < 0.001) No statistically significant difference was found in BMI between the groups of patients with CP and those with comorbid OA and CP (**Supplementary Table S2**).

The study revealed no significant differences in the levels of RBC (p = 0.0688), WBC (p = 0.065), and hemoglobin (p = 0.3282) across the groups.

Differences in iron and ferritin levels were observed across all studied groups (p < 0.001). Patients with OA and CP exhibited lower levels of iron and ferritin compared to the control group (p < 0.001). Furthermore, those with comorbid OA and CP without comorbidity showed lower indicator levels than those with the OA group (p < 0.001). Diverse levels of transferrin were also identified among all examined groups (p < 0.001). No statistically significant difference was found in transferrin levels between the groups of patients with CP and with comorbid OA and CP. Patients with OA showed lower levels of this biomarker compared to the comorbidity group, yet higher levels compared to the control group (p < 0.001) (**Supplementary Table S2**).

Analysis of CRP levels revealed distinct variations among all investigated groups (p < 0.001). The highest levels of CRP were detected in the group of patients with comorbid OA and CP (p < 0.001). Within the CP group and OA group CRP levels were lower compared to the comorbidity group (p < 0.001) but higher compared to the control group (p < 0.001). However, the CRP level remained within the reference range (**Supplementary Table S2**).

### 3.2 Correlation analysis of data in patients with OA and OA+CP

**Figure 1:**
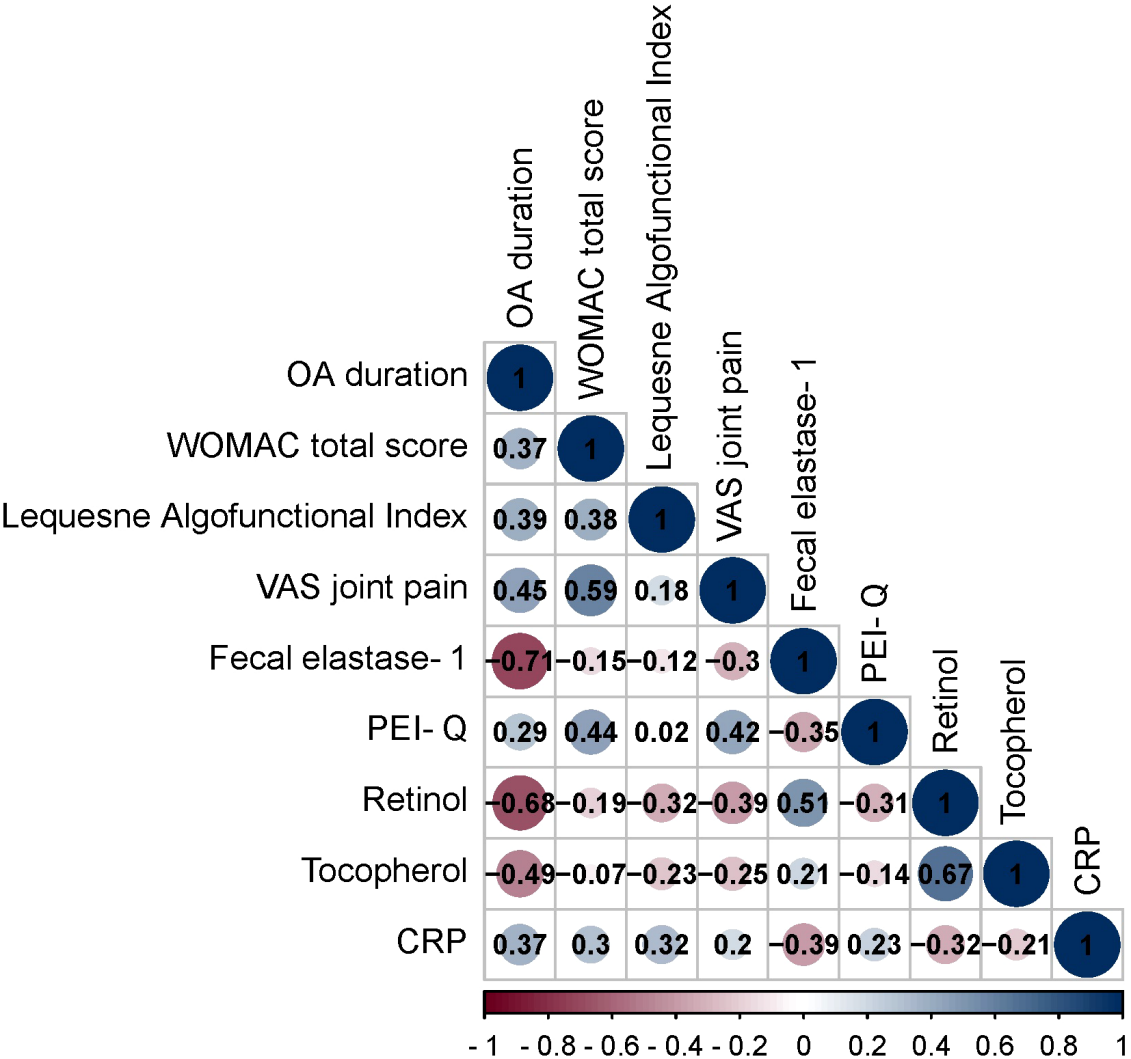
Spearman correlation correlogram used for correlations between data in patients with OA. Red: Strong negative correlation (r = −1.0). Blue: Strong positive correlation (r = 1.0).

This section examines the correlations between various clinical parameters in OA patients without CP. The analysis utilized Spearman’s rank correlation coefficient (r) to evaluate the strength and direction of these associations (**Figure 1**).

The OA duration demonstrated positive and negative correlations with multiple parameters. These correlations encompassed WOMAC total score (r = 0.37, p = 0.002), Lequesne Algofunctional Index (r = 0.39, p = 0.0009), VAS joint pain (r = 0.45, p = 0.0001), fecal elastase-1 (r = -0.71, p < 0.001), PEI-Q (r = 0.29, p = 0.016), retinol (r = -0.68, p < 0.001), tocopherol (r = -0.49, p < 0.001), CRP (r = 0.37, p = 0.002).

The WOMAC total score exhibited positive correlations with Lequesne Algofunctional Index (r = 0.38, p = 0.002), VAS joint pain (r = 0.59, p < 0.001), PEI-Q (r = 0.44, p = 0.0002), CRP (r = 0.30, p = 0.012).

The Lequesne Algofunctional Index showed positive correlations with retinol (r = -0.32, p = 0.007) and CRP (r = 0.32, p = 0.007).

The VAS joint pain displayed positive and negative correlations with fecal elastase-1 (r = -0.30, p = 0.011), PEI-Q (r = 0.42, p = 0.0003), retinol (r = -0.39, p = 0.0003).

The fecal elastase-1 exhibited positive and negative correlations with PEI-Q (r = -0.35, p = 0.0039), retinol (r = 0.51, p < 0.001), and CRP (r = -0.39, p = 0.001).

The PEI-Q showed a negative correlation with retinol (r = -0.31, p = 0.009).

The retinol demonstrated positive and negative correlations with tocopherol (r = 0.67, p < 0.001), CRP (r = -0.32, p = 0.007).

**Figure 2:**
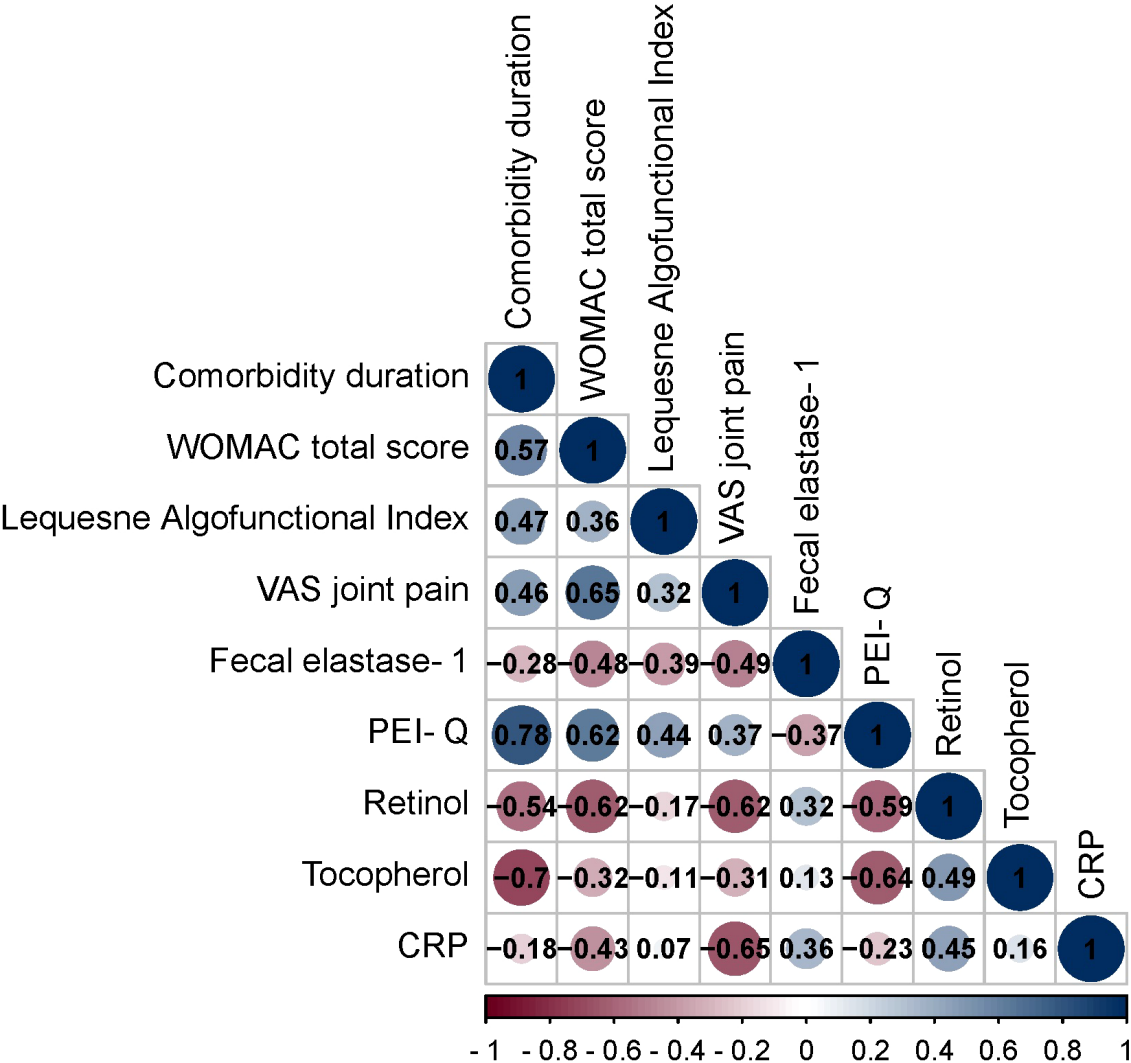
Spearman correlation correlogram used for correlations between data in patients with OA+CP. Red: Strong negative correlation (r = −1.0). Blue: Strong positive correlation (r = 1.0).

The following section investigates the correlations between diverse clinical parameters in OA patients with CP. Spearman’s rank correlation coefficient (r) was employed to assess the magnitude and direction of these relationships (**Figure 2**).

The duration of OA exhibited both positive and negative correlations with various parameters. These correlations included the WOMAC total score (r = 0.57, p < 0.001), Lequesne Algofunctional Index (r = 0.47, p = 0.0005), VAS joint pain (r = 0.46, p = 0.0005), fecal elastase-1 (r = -0.28, p = 0.04), PEI-Q (r = 0.78, p < 0.001), retinol (r = -0.54, p < 0.001), tocopherol (r = -0.70, p < 0.001).

The WOMAC total score displayed positive and negative correlations with Lequesne Algofunctional Index (r = 0.36, p = 0.008), VAS joint pain (r = 0.65, p < 0.001), fecal elastase-1 (r = -0.48, p = 0.0004), PEI-Q (r = 0.62, p < 0.001), retinol (r = -0.62, p < 0.001), tocopherol (r = -0.32, p = 0.019) and CRP (r = -0.43, p = 0.001).

The Lequesne Algofunctional Index showed positive and negative correlations with VAS joint pain (r = 0.32, p = 0.022), fecal elastase-1 (r = -0.39, p = 0.004), PEI-Q (r = 0.44, p = 0.0009).

The VAS joint pain exhibited positive and negative correlations with fecal elastase-1 (r = -0.49, p = 0.0003), PEI-Q (r = 0.37, p = 0.007), retinol (r = -0.62, p < 0.001), tocopherol (r = -0.31, p = 0.025) and CRP (r = -0.65, p < 0.001).

The fecal elastase-1 demonstrated positive and negative correlations with PEI-Q (r = -0.37, p = 0.006), retinol (r = 0.32, p = 0.02), CRP (r = 0.36, p = 0.0098).

The PEI-Q showed negative correlations with retinol (r = -0.59, p < 0.001) and tocopherol (r = -0.64, p < 0.001).

The retinol exhibited positive correlations with tocopherol (r = 0.49, p = 0.0003) and CRP (r = 0.45, p = 0.00008).

### 3.3 Predictors analysis of OA and CP comorbidity and EPI

**Figure 3:**
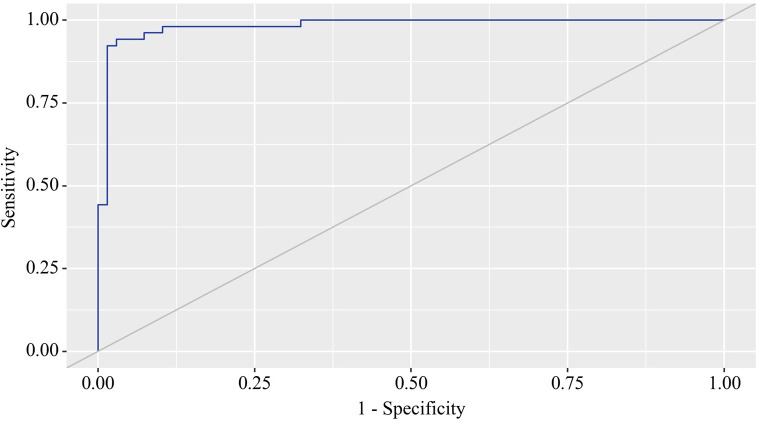
ROC curve characterizing the relationship between BMI and the comorbidity formation of OA and CP.

Using the ROC analysis, we examined predictors influencing the comorbidity formation of OA and CP. BMI emerged as a significant predictor of this comorbidity formation. The area under the ROC curve was 0.983 ± 0.011, with a 95% confidence interval ranging from 0.960 to 1.000. The generated model demonstrated statistical significance (p < 0.001) (**Figure 3**).

**Figure 4:**
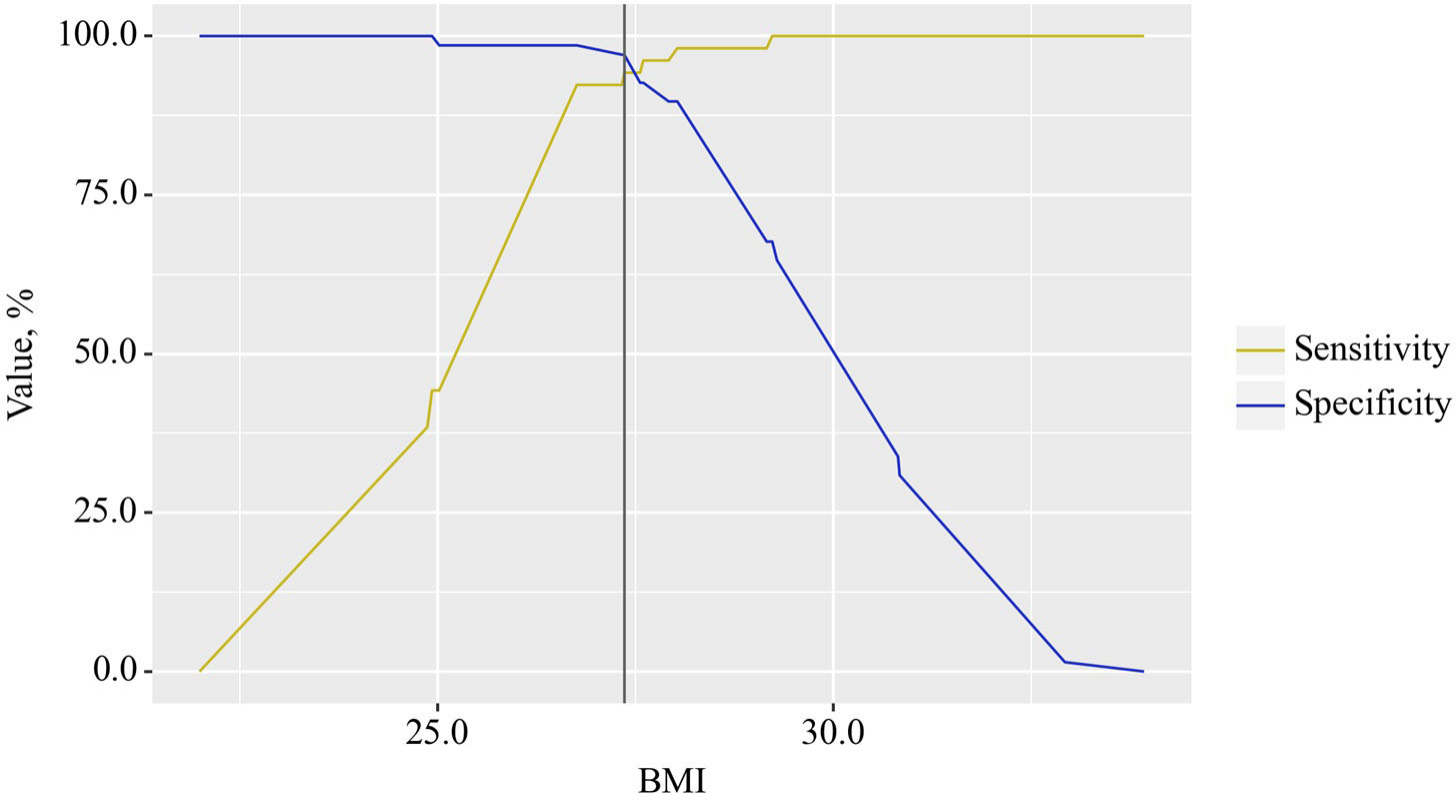
Analysis of the sensitivity and specificity of the relationship between BMI on the comorbidity formation of OA and CP.

The BMI cut-off value corresponding to the highest Youden’s J statistic was determined to be 27.360. Predictive outcomes indicated that BMI values below this threshold were associated with comorbidity of OA and CP. The method’s sensitivity and specificity were calculated as 94.2% and 97.1%, respectively (**Figure 4**). Predictive outcomes indicated that BMI values below this threshold were associated with comorbidity of OA and CP. A decrease in BMI below this value can be utilized in clinical practice as a predictor of the presence of comorbidity between OA and CP in patients, with high sensitivity and specificity, ensuring good reliability of the results.

**Figure 5:**
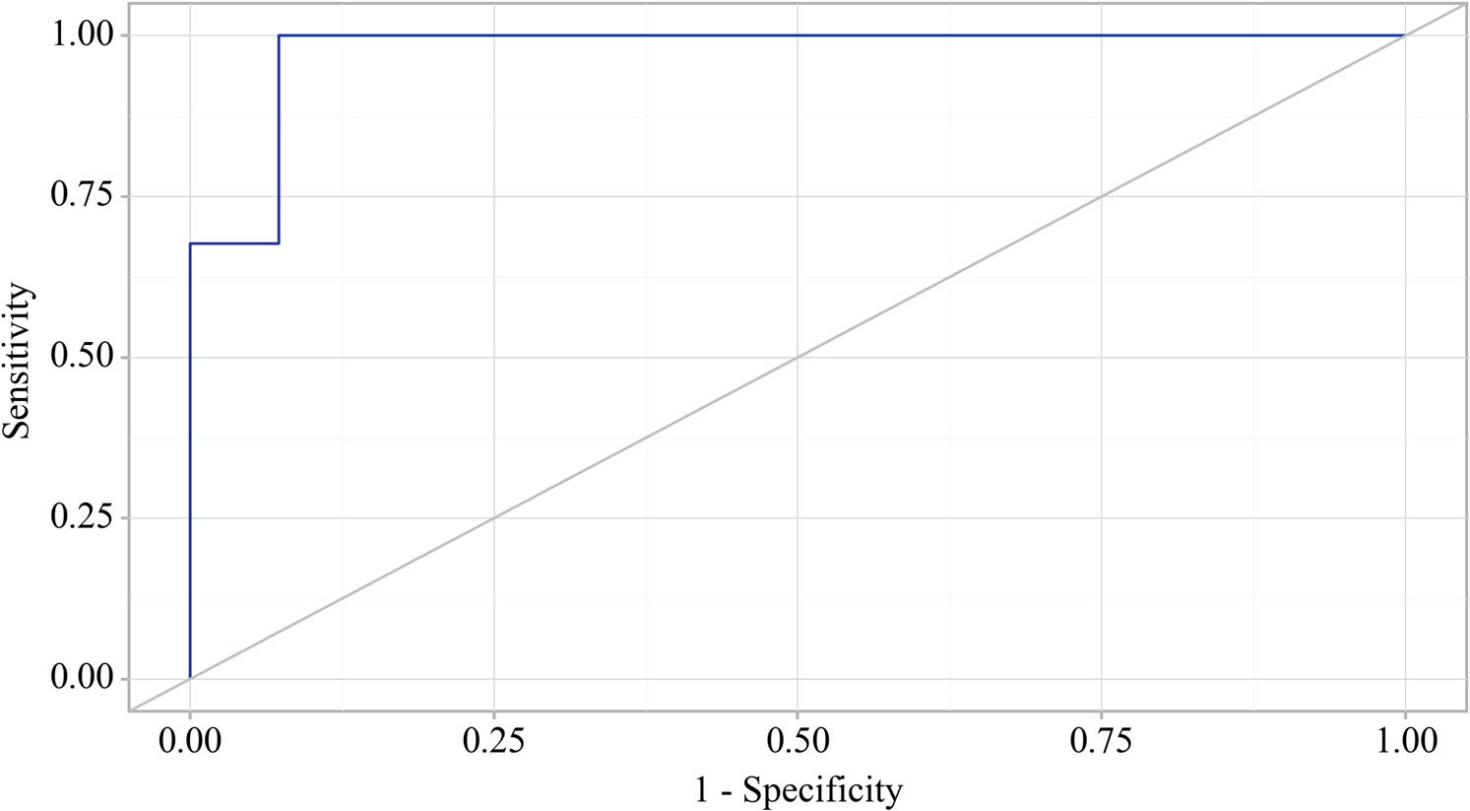
ROC curve characterizing the relationship between fecal elastase-1 levels and the formation of exocrine pancreatic insufficiency (EPI) in OA.

In the study of EPI predictors in patients with OA, ROC analysis identified that a reduction in fecal elastase-1 also indicates the presence of EPI in patients with osteoarthritis (**Figure 5**).

The area under the ROC curve was 0.976 ± 0.012, with a 95% confidence interval of 0.952 to 1.000. The generated model showed statistical significance (p < 0.001).

**Figure 6:**
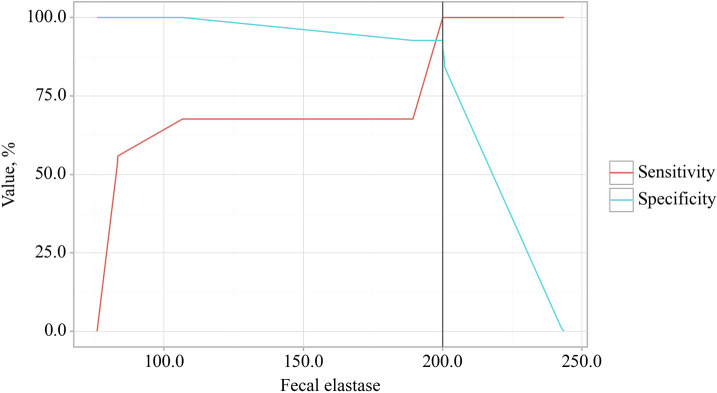
Analysis of the sensitivity and specificity regarding the association between fecal elastase-1 levels and the development of exocrine pancreatic insufficiency (EPI) in OA.

The cut-off value for fecal elastase corresponding to the highest Youden’s J statistic was determined to be 200.060. Predictions indicated that fecal elastase-1 levels below this threshold were associated with EPI. The method’s sensitivity and specificity were calculated as 100.0% and 92.7%, respectively (**Figure 6**). A reduction in fecal elastase-1 levels below this threshold may serve as a valuable clinical predictor of the development of chronic pancreatitis with exocrine pancreatic insufficiency in patients with osteoarthritis, supported by high sensitivity and specificity that confer strong reliability to the findings.

### 3.4 Principal component analysis

**Figure 7:**
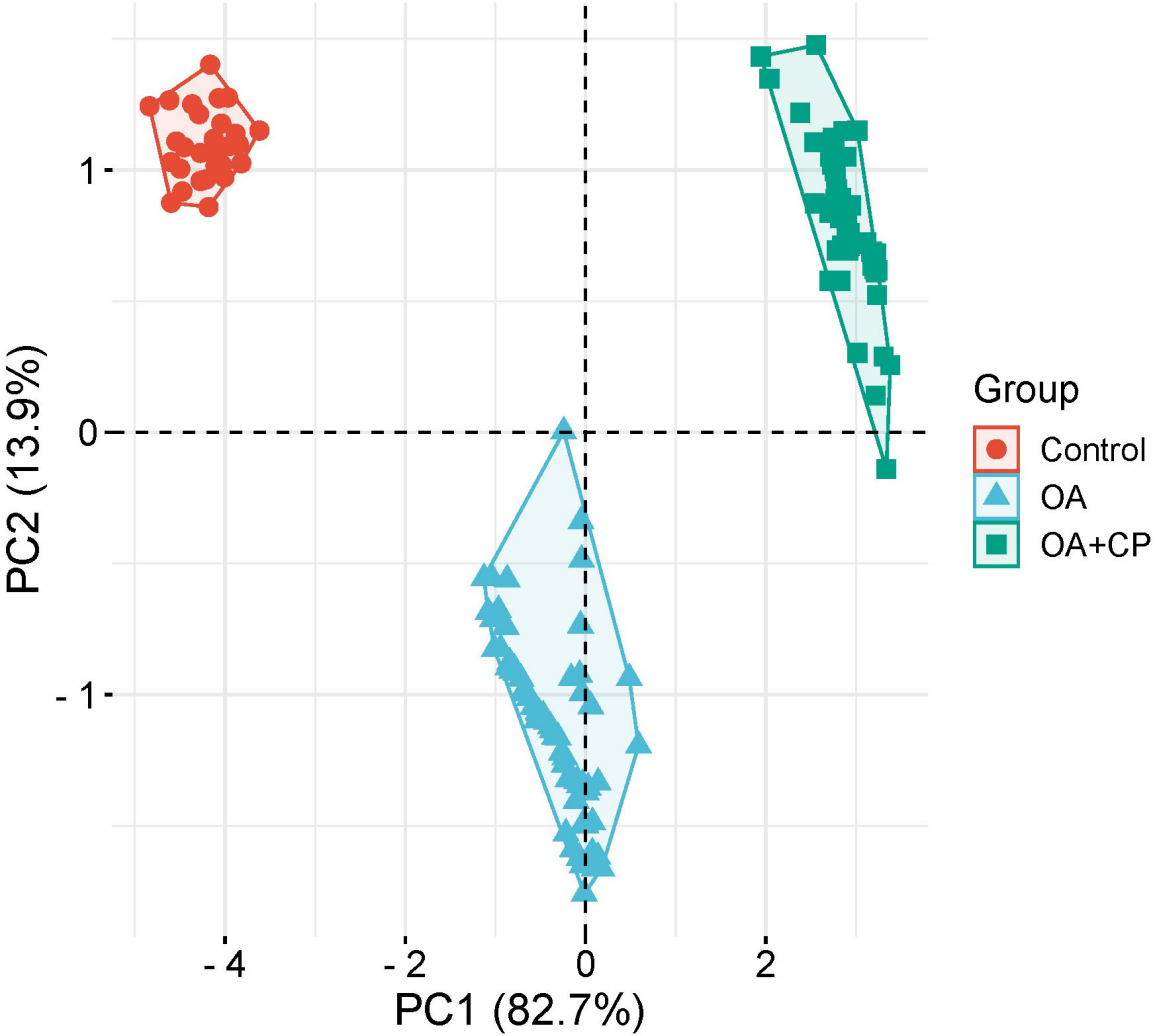
Principal component analysis for the OA and comorbidity presence.

Principal component analysis (PCA) was employed to investigate the underlying determinants contributing to the OA and comorbidity presence (**Figure 7**). The PCA identified two principal components (PCs) related to the OA and comorbidity presence, accounting for 96.6% of the variance (82.7% by PC1 and 13.9% by PC2). The Kaiser–Meyer–Olkin (KMO) measure of sampling adequacy was 0.889, indicating the correlation matrix was suitable for PCA. Bartlett’s test of sphericity showed a significance level of p < 0.001, further confirming the appropriateness of using PCA.

PC1 included the WOMAC total score, fecal elastase-1, CRP, ferritin, retinol, tocopherol, and 25(OH) vitamin D3. PC2 comprised BMI (**Table 3**).

**Table 3:** Rotated component matrix for the OA and comorbidity presence.

	Component	
	Factor 1	Factor 2
WOMAC total score	-0.890	0.429
Fecal elastase-1	0.976	0.172
CRP	-0.979	0.104
BMI	-0.110	0.992
Ferritin	0.978	-0.135
Retinol	0.920	-0.227
Tocopherol	0.947	-0.289
25(OH) vitamin D3	0.957	-0.187

Extraction Method: Principal Component Analysis. Rotation Method: Varimax with Kaiser Normalization. Rotation converged in 3 iterations.

The PCA findings illustrate that a combination of inflammatory markers, nutritional status, and body composition are critical determinants of the comorbidity between osteoarthritis (OA) and chronic pancreatitis. This understanding can assist clinicians in developing targeted interventions that address both the physical and systemic aspects of OA, ultimately improving patient outcomes.

### 3.5 Binary logistic regression

Using binary logistic regression, a predictive model was constructed to predict the likelihood of EPI presence in OA based on GSRS-DS, GSRS-CS, PEI-Q-A, Retinol, Tocopherol, and Iron. The dataset comprised 150 observations. The relationship observed can be expressed by the following equation:


(1) $$\text{P}=1/\left(1+{\text{e}}^{-z}\right)\times 100\%,$$

(2) $$\begin{array}{l} \text{z}=-0.379+2.027{\text{X}}_{\text{GSRS}-\text{DS}}-5.942{\text{X}}_{\text{GSRS}-\text{CS}}+10.440{\text{X}}_{\text{PEI}-\text{Q}-\text{A}}-17.074{\text{X}}_{\text{Retinol}}+\\ 1.732{\text{X}}_{\text{Tocopherol}}-0.549{\text{X}}_{\text{Iron}} \end{array}$$

where P – probability of m-mod-s, X_GSRS-DS_ – GSRS-DS, X_GSRS-CS_ – GSRS-CS, X_PEI-Q-A_ – PEI-Q-A, X_Retinol_ – Retinol, X_Tocopherol_ – Tocopherol, X_Iron_ – Iron

The resulting regression model is statistically significant (p < 0.001). According to the Nagelkerke R² value, the model accounts for 77.0% of the variability observed in EPI presence in OA.

**Figure 8:**
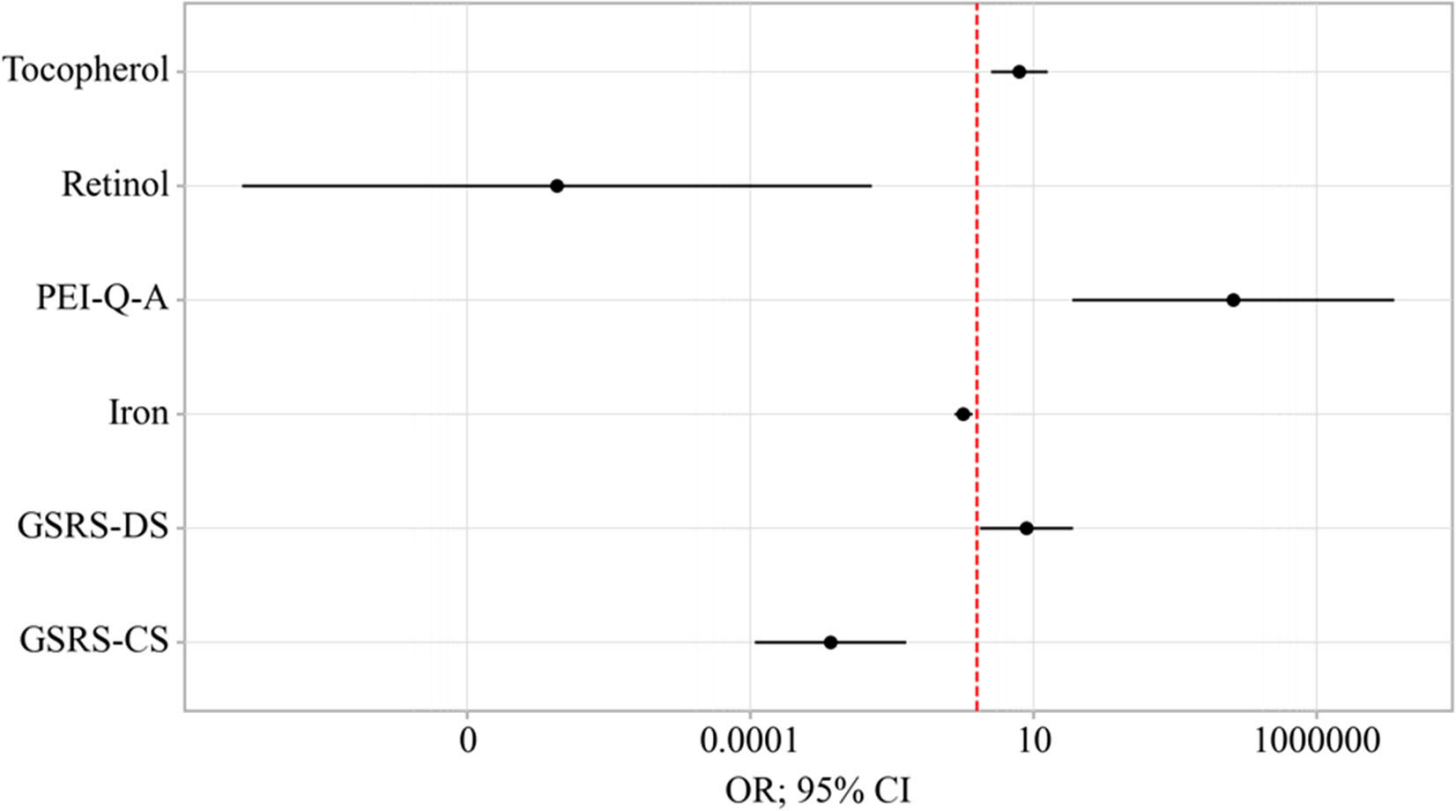
Odds ratios estimates with corresponding 95% CI’s for predictors included to the model EPI presence in OA.

An increase in GSRS-DS is associated with a 7.592-fold increase in the odds of EPI presence in OA. Conversely, an increase in GSRS-CS is associated with a 380.696-fold decrease in the odds of EPI presence in OA. An increase in PEI-Q-A corresponds to a 34,207.493-fold increase in the odds of EPI presence in OA. On the other hand, an increase in Retinol is associated with a 26,002,415.617-fold decrease in the odds of EPI presence in OA. A one-unit increase in Tocopherol is linked with a 5.651-fold increase in the odds of EPI presence in OA, while a one-unit increase in Iron is associated with a 1.732-fold decrease in the odds of EPI presence in OA (**Table 4**) (**Figure 8**).

**Table 4:** Characteristics of the association of predictors with the probability of EPI presence in OA.

Predictors	Unadjusted		Adjusted	
	COR; 95% CI	p	AOR; 95% CI	p
GSRS-DS	2.773; 2.061 – 3.732	< 0.001*	7.592; 1.146 – 50.350	0.036*
GSRS-CS	2.511; 1.916 – 3.294	< 0.001*	0.003; 0.000 – 0.057	< 0.001*
PEI-Q-A	10.285; 5.186 – 20.389	< 0.001*	34207.493; 48.570 – 24082596.484	0.002*
Retinol	0.000; 0.000 – 0.000	< 0.001*	0.000; 0.000 – 0.014	0.009*
Tocopherol	0.399; 0.296 – 0.538	< 0.001*	5.651; 1.793 – 17.814	0.003*
Iron	0.705; 0.634 – 0.784	< 0.001*	0.577; 0.399 – 0.835	0.004*

*association of the outcome value with the predictor value is statistically significant (p < 0.05).

**Figure 9:**
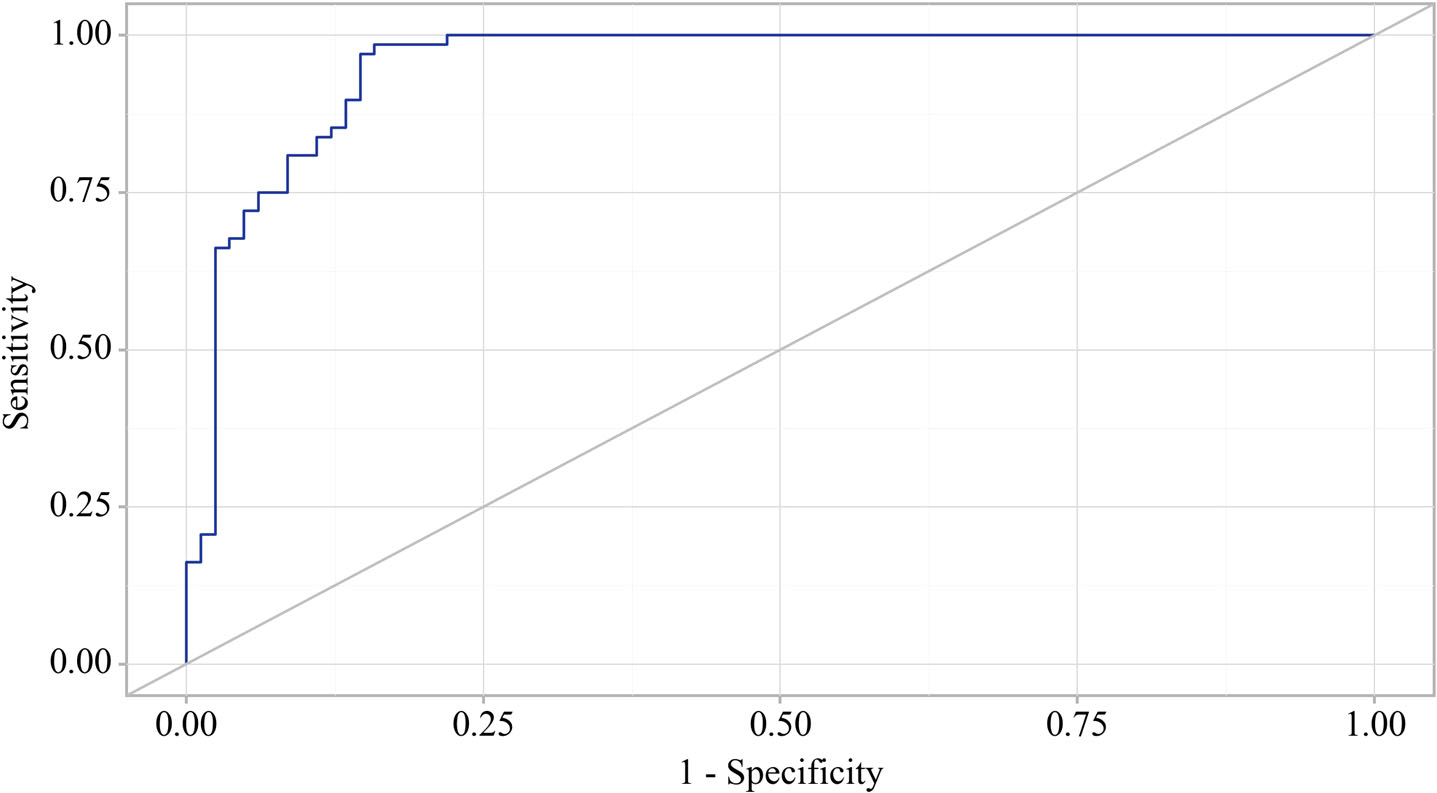
ROC-curve characterizing the dependence of the probability EPI presence in OA on Value of logistic function P.

The resulting curve was obtained when assessing the relationship between the probability of EPI presence in OA and the value of the logistic function P using ROC analysis (**Figure 9**).

The area under the ROC curve was 0.952 ± 0.019 (95% CI: 0.914 - 0.989). The resultant model was statistically significant (p < 0.001).

**Figure 10:**
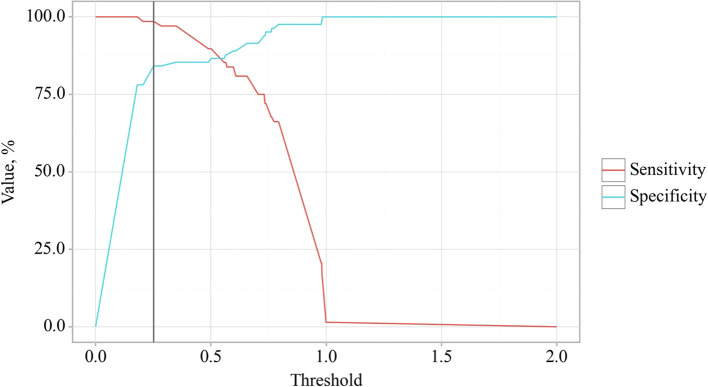
Analysis of the sensitivity and specificity of EPI presence in OA depending on Value of logistic function P.

The cut-off value of the logistic function P, which maximizes Youden’s J statistic, was determined to be 0.252. When the logistic function P was more significant than or equal to this threshold, EPI presence in OA was predicted. The method’s sensitivity and specificity were 98.5% and 84.1%, respectively (**Figure 10**).

## 4 Discussion

**Figure 11:**
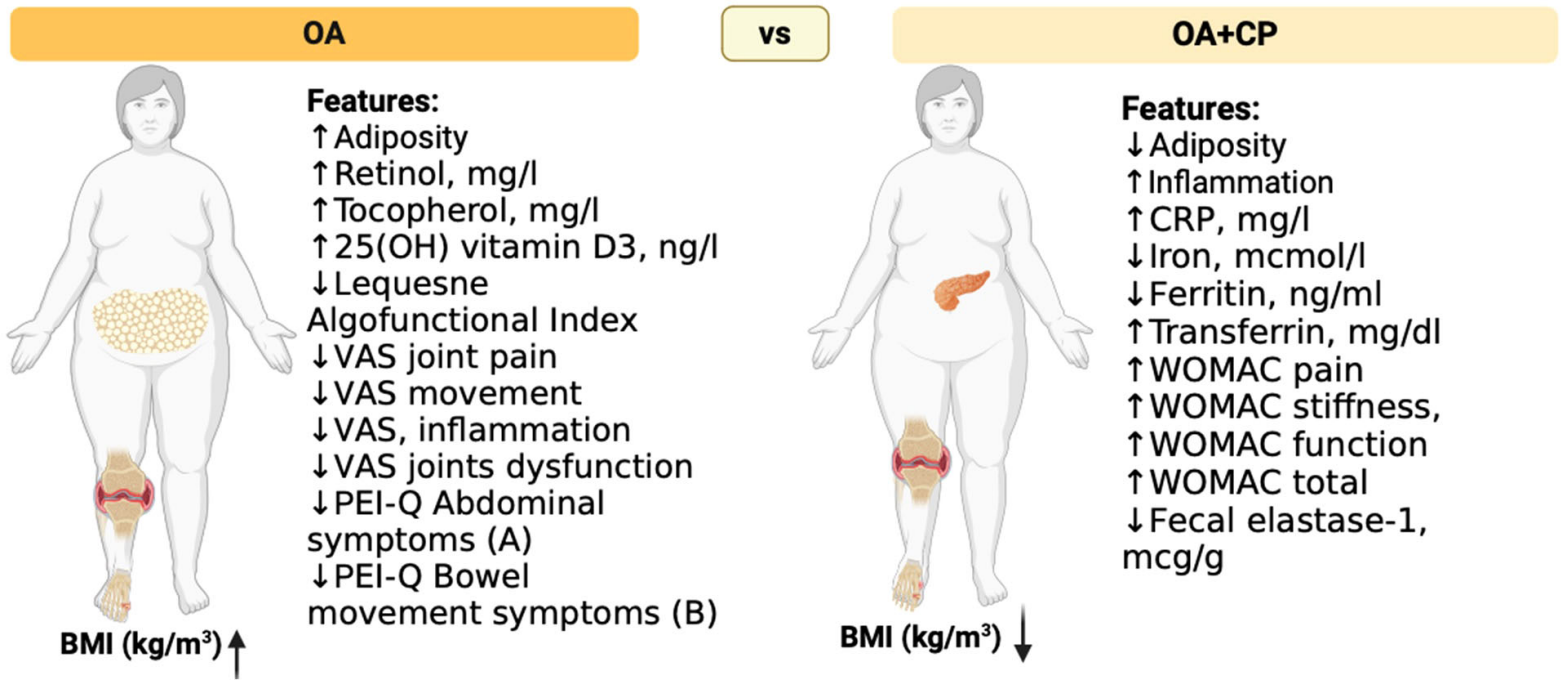
Clinical and laboratory signs of comorbid course of OA and CP. The figure illustrates notable differences between osteoarthritis (OA) patients and those with both osteoarthritis and chronic pancreatitis (OA+CP) in terms of adiposity, inflammation markers, nutrient levels, and functional indices. According to our study, OA+CP patients exhibit higher inflammation markers, lower iron levels, higher ferritin and transferrin levels, and worse WOMAC scores for pain, stiffness, and functional activity. Additionally, these patients have decreased vitamin levels. The Figure was designed using BioRender.

This study explored the effects of comorbidity between osteoarthritis (OA) and chronic pancreatitis (CP) on the progression and clinical course of these conditions. The findings indicated that the presence of comorbidity between OA and CP negatively impacts disease indicators compared to patients who do not have this comorbidity. Key potential predictors for the development of comorbidity between osteoarthritis and chronic pancreatitis included Body Mass Index (BMI), the total score of the Western Ontario and McMaster Universities Osteoarthritis Index (WOMAC), fecal elastase-1 levels, C-reactive protein (CRP), ferritin, retinol, tocopherol, 25(OH) vitamin D3, and various gastrointestinal symptom assessment scales (**Figure 11**).

A prior study hypothesized that CP leads to alterations in articular cartilage and subchondral bone, potentially contributing to the development of OA. Findings revealed significant changes in subchondral bone and articular cartilage, including reduced bone volume and decreased proteoglycan content, suggesting a link between CP and musculoskeletal alterations. This highlights the need for further research into the systemic effects of CP (29, 30). Recent advances in understanding the molecular mechanisms of inflammation in OA have highlighted the complexity of cellular contributions to joint destruction and tissue regeneration, emphasizing the need for clinical trials on anti-inflammatory treatments, especially sustained-release intra-articular formulations, to mitigate disease progression and associated pain (31). Advances in understanding how damage-associated molecular patterns (DAMPs) trigger monocyte/macrophage recruitment and activation in joints offer potential for disease-modifying therapies, but the complexity of macrophage phenotypes and their distinct cellular lineages has hindered treatment development, highlighting the need for precise patient selection based on specific OA subtypes like obesity or genetic risk (32). In our study, it was also established that the course of OA is worse in the presence of comorbidity with CP, which indicates the potential influence of CP on the progression of OA, which is consistent with resent studies and requires further investigation.

OA is now established as a low-grade inflammatory disease that affects the entire joint, with synovial macrophages playing a crucial role in its symptomatology and progression through various signaling pathways that regulate their activation and polarization. This underscores the potential of macrophage reprogramming from the M1 to M2 phenotype as a promising therapeutic strategy (33). Cartilage lesions and OA present a growing clinical and socioeconomic burden, primarily driven by synovial inflammation and an inflammatory articular environment that contribute to chondrocyte apoptosis and hypertrophy, ectopic bone formation, and OA progression.

Effective treatment of OA necessitates the development of therapeutic agents that shift inflammation towards a pro-chondrogenic microenvironment, with promising approaches including immune cell modulation and cell therapy, particularly through the promotion of anti-inflammatory M2 macrophages via various stimuli such as physical exercise and mesenchymal stem cells (MSCs) (34). Findings suggest a potential positive correlation between higher serum C reactive protein (CRP) levels and increased pain sensitivity, more research is needed (35).

Researches also highlight the pivotal role of inflammation in CP. An elevation in CRP levels may increase the risk of developing pancreatic cancer in the future, as elevated levels of these biomarkers are associated with worse survival outcomes in patients with CP (36). Macrophages play a critical role in the pathogenesis of pancreatitis. They exhibit distinct phenotypic variations and functions in acute pancreatitis (AP) and CP. In CP, macrophages tend to polarize towards an M2 phenotype, interacting with pancreatic stellate cells (PSCs) through autocrine and paracrine cytokine signaling, thereby promoting pancreatic fibrosis progression (37). Macrophage-derived cytokines serve as biomarkers offering new avenues for early diagnosis CP and differentiation from pancreatic cancer and other pancreatic disorders. In established CP, interactions between macrophages and T lymphocytes contribute to immune dysregulation, with macrophage-produced proinflammatory cytokines playing a critical role in driving acinar-to-ductal metaplasia (ADM) (38). An in-depth exploration of how inflammatory mediators, including CRP, IL-6, TNF-α, and fibrinogen, contribute to pancreatic dysfunction and their interaction with OA would significantly enhance our mechanistic understanding of comorbidity. The chronic inflammatory state associated with OA may further exacerbate pancreatic damage by fostering insulin resistance and modifying the local microenvironment of the pancreas, as confirmed by previous studies (31, 39).

The study demonstrated that CP was associated with gut dysbiosis, characterized by a reduced abundance of Gram-positive (G+) bacteria that produce short-chain fatty acids (SCFAs). Specifically, the depletion of G+ bacteria exacerbated the severity of CP, highlighting their critical role in modulating pancreatic fibrosis (40, 41). Dysbiosis, alongside metabolic factors such as hyperinsulinemia, insulin resistance, dyslipidemia, overstimulation of the sympathetic nervous system and renin-angiotensin system, and oxidative stress, leads to dysfunction of the gut barrier, increased intestinal permeability, and the release of toxic bacterial metabolites into circulation (42–44). It was indicated that alterations in gut microbiota could serve as a potential biomarker for inflammation, as measured by CRP levels, in patients with T2D and COVID-19 (45). These factors collectively promote the development of low-grade systemic inflammation (46).

Recent research has revealed specific alterations in the microbiome linked to OA, characterized by an elevated Firmicutes/Bacteroides ratio, increased prevalence of Streptococcus spp., and localized inflammatory responses (47). The gastrointestinal tract represents a compelling and innovative target for OA therapy (47).

In our investigation, we detected an inflammatory condition in individuals diagnosed with OA, including those concurrently diagnosed with pancreatitis. Moreover, our findings revealed that this comorbidity was associated with higher C-reactive protein levels, suggesting heightened inflammatory activity. These results are consistent with existing studies (48–51), there remains an insufficient body of research concerning the inflammatory impact of OA and CP comorbidity on disease progression and complication development, necessitating further investigation. In future studies, we plan to explore additional inflammatory markers to gain a more comprehensive understanding of this comorbidity.

Non-steroidal anti-inflammatory drugs (NSAID) are frequently utilized pharmacological agents in the management of pain associated with OA (52, 53). However, the administration of these drugs is linked with substantial gastrointestinal toxicity, impacting both the upper gastrointestinal tract, potentially causing peptic ulcer disease, and the lower gastrointestinal tract, which can result in NSAID-induced enteropathy. Furthermore, NSAID usage has been associated with an increased likelihood of clinical relapse in individuals with inflammatory bowel disease, underscoring the necessity for cautious prescribing in this patient population (54–56). This damage is attributed to mechanisms involving prostaglandin-endoperoxide synthase 1 (PTGS1 or COX1) and PTGS2 (COX2), as well as additional factors (57, 58). These mechanisms include NSAID interactions with phospholipids and the uncoupling of mitochondrial oxidative phosphorylation, which lead to the disruption of gastrointestinal barrier function, increased intestinal permeability, and low-grade inflammation (57, 58).

The inhibition of COX enzymes by NSAIDs, coupled with the presence of luminal aggressors, results in the formation of erosions and ulcers, with potential complications such as bleeding, protein loss, stricture formation, and perforation (59). Damage to the pancreatic systems caused by non-steroidal anti-inflammatory drugs (NSAIDs) may present as pancreatitis (60, 61). The occurrence of these complications may result in digestive disorders, impaired nutrient absorption, and subsequently, the onset of malnutrition (62, 63). Exocrine pancreatic insufficiency associated with CP contributes to the development of malnutrition (18, 64). In our investigation, we also documented gastrointestinal symptomatology in patients with OA evaluated by the GSRS scale, potentially exacerbated by long-term NSAID use, consistent with previous research. Furthermore, we observed the onset of worsening malnutrition based on levels of fat-soluble vitamins and markers of iron metabolism, particularly intensified in the presence of comorbid OA and CP compared to patients with CP without the studied comorbidity, which warrants further research.

## 5 Limitations

We recognize that our study possesses several limitations. Primarily, the relatively small sample size restricts the generalizability of our results to a broader population. A more extensive multicenter study would be required to validate these findings and improve their generalizability. Additionally, the monocentric design of this research inherently limits the studied population and may introduce selection bias. Future studies should ideally include participants from multiple centers to obtain a more representative sample. Furthermore, the investigated parameters were not analyzed in a cohort of patients with CP who do not have comorbid OA. In the perspective of further research, we plan to study other parameters that can potentially affect the course of osteoarthritis through inflammation and other pathogenetic mechanisms.

## 6 Conclusions

In this research, the study focused on exploring the influence of the comorbidity of CP and OA on the progression of these diseases. It was determined that the presence of comorbidity between OA and CP potentially exacerbates the progression of both conditions, as indicated by the studied markers, compared to patients who have osteoarthritis and chronic pancreatitis without this comorbidity. Potential predictors of OA and CP comorbidity and the development of complications emerged as BMI, the total score on the Western Ontario and McMaster Universities Osteoarthritis Index (WOMAC), fecal elastase-1, C-reactive protein (CRP), ferritin, retinol, tocopherol, and 25-hydroxyvitamin D3. Furthermore, the Gastrointestinal Symptom Rating Scale-Diarrhea Syndrome (GSRS-DS), the Gastrointestinal Symptom Rating Scale-Constipation Syndrome (GSRS-CS), and the Qualitative Assessment of the Symptoms and Impact of Pancreatic Exocrine Insufficiency Domain A (PEI-Q-A), along with retinol, tocopherol, and iron, were recognized as potential predictors for the presence of exocrine pancreatic insufficiency in patients with OA. The clinical applicability of our findings is emphasized by the potential for monitoring BMI or CRP levels in patients with osteoarthritis, as this strategy could assist in preventing complications associated with the comorbidity of osteoarthritis and chronic pancreatitis. By identifying potential predictors of this comorbidity and its complications, we lay the foundation for future research that may enhance clinical strategies for managing patients with these conditions.

## Data Availability

The raw data supporting the conclusions of this article will be made available by the authors, without undue reservation.
